# Dietary patterns within educational groups and their association with CHD and stroke in the European Prospective Investigation into Cancer and Nutrition-Netherlands cohort

**DOI:** 10.1017/S0007114518000569

**Published:** 2018-04-28

**Authors:** Sander Biesbroek, Mirjam C. Kneepkens, Saskia W. van den Berg, Heidi P. Fransen, Joline W. Beulens, Petra H. M. Peeters, Jolanda M. A. Boer

**Affiliations:** 1 National Institute for Public Health and the Environment, Antonie van Leeuwenhoeklaan 9, 3721 MA Bilthoven, The Netherlands; 2 Julius Center for Health Sciences and Primary Care, University Medical Center Utrecht, Universiteitsweg 100, 3584 CG Utrecht, The Netherlands; 3 Department of Epidemiology & Biostatistics, EMGO+ Institute for Health and Care Research, VU University Medical Center, De Boelelaan 1105, 1081 HV Amsterdam, The Netherlands; 4 School of Public Health, Imperial College London, South Kensington Campus, London SW7 2AZ, UK

**Keywords:** Socio-economic status, Dietary patterns, CVD, Principal component analysis, Education

## Abstract

Higher-educated people often have healthier diets, but it is unclear whether specific dietary patterns exist within educational groups. We therefore aimed to derive dietary patterns in the total population and by educational level and to investigate whether these patterns differed in their composition and associations with the incidence of fatal and non-fatal CHD and stroke. Patterns were derived using principal components analysis in 36 418 participants of the European Prospective Investigation into Cancer and Nutrition-Netherlands cohort. Self-reported educational level was used to create three educational groups. Dietary intake was estimated using a validated semi-quantitative FFQ. Hazard ratios were estimated using Cox Proportional Hazard analysis after a mean follow-up of 16 years. In the three educational groups, similar ‘Western’, ‘prudent’ and ‘traditional’ patterns were derived as in the total population. However, with higher educational level a lower population-derived score for the ‘Western’ and ‘traditional’ patterns and a higher score on the ‘prudent’ pattern were observed. These differences in distribution of the factor scores illustrate the association between education and food consumption. After adjustments, no differences in associations between population-derived dietary patterns and the incidence of CHD or stroke were found between the educational groups (*P*_interaction_ between 0·21 and 0·98). In conclusion, although in general population and educational groups-derived dietary patterns did not differ, small differences between educational groups existed in the consumption of food groups in participants considered adherent to the population-derived patterns (Q4). This did not result in different associations with incident CHD or stroke between educational groups.

It is well established that there are socio-economic inequalities in health^(^ ^1^ ^)^. Life expectancy at birth ranges from 46 years in a poor country like Sierra Leone up to 84 years in a wealthy country like Japan^(^ ^2^ ^)^. This inequality is also seen within countries. In the Netherlands, life expectancy is 6 years longer for people who attended higher vocational education or university compared with people who only attended primary school^(^ ^3^ ^)^. In almost all countries, morbidity and mortality risks are higher in groups of lower socio-economic status (SES)^(^ ^4^ ^,^ ^5^ ^)^. For example, stroke, diseases of the nervous system, diabetes and arthritis show relatively large inequalities (OR >1·50) across eight European countries^(^ ^6^ ^)^.

Health inequalities are partly due to differences in health behaviours between socio-economic groups^(^ ^7^ ^)^. The socio-economic inequalities in health behaviours, such as dietary habits, are substantial^(^ ^8^ ^)^. Diet is linked to a large number of health outcomes, and an improved dietary intake can help reduce the risk of many diseases – for example, CHD and stroke^(^ ^9^ ^,^ ^10^ ^)^. Dietary patterns that are characterised by a high consumption of fruit, vegetables, whole grains, fish and poultry and a low consumption of meat and refined grains are seen as healthy and are associated with a more adequate intake of nutrients and lower energy density^(^ ^11^ ^)^. Such healthy dietary patterns are more frequently observed in groups of high SES^(^ ^11^ ^,^ ^12^ ^)^. The low cost of energy-dense foods may mediate the association between education and obesity^(^ ^13^ ^)^, a known risk factor for CVD^(^ ^14^ ^)^. Therefore, improving diet may contribute to the reduction in socio-economic health inequalities.

However, a healthy diet can be achieved in many ways, and healthy food choices may differ according to SES. If distinct dietary patterns exist across SES groups, then policy, education and communication strategies to increase health in lower socio-economic groups could be targeted to these specific patterns. This may reduce the gap in health inequalities^(^ ^15^ ^)^. Current studies that examined the association between *a posteriori* dietary patterns and SES identified the patterns based on the whole population and thereafter assessed the relationship with SES^(^ ^5^ ^,^ ^16^ ^–^ ^18^ ^)^. By doing so, they assume that the underlying patterns are the same for different SES groups. However, this may not be the case. In addition, differences in intake might lead to different associations between dietary patterns and the incidence of CHD/stroke.

We aim to investigate in a Dutch cohort whether *a posteriori* dietary patterns based on the whole population differ from dietary patterns derived for different SES groups (based on educational attainment). Furthermore, we evaluate the food intake in adherents of the obtained dietary patterns and the association of the dietary patterns with the incidence of fatal and non-fatal CHD and stroke.

## Methods

### Study population

For this study, data of the European Prospective Investigation into Cancer and Nutrition-Netherlands (EPIC-NL) cohort were used. This cohort is the Dutch contribution to the EPIC. The cohort consists of the EPIC-Prospect cohort (17 357 women aged 50–69 years at baseline) and the EPIC-MORGEN cohort (22 654 men and women aged 20–64 years at baseline). EPIC-Prospect is a study among women residing in the city of Utrecht or its vicinity, who participated in the nationwide Dutch breast cancer screening programme. In the EPIC-MORGEN cohort, participants from Amsterdam, Maastricht and Doetinchem were included through random population sampling. All participants were recruited between 1993 and 1997 and gave written informed consent before the study^(^ ^19^ ^)^. Both cohorts comply with the Declaration of Helsinki.

From the initial 40 011 participants enrolled in the study, participants were excluded if educational level was missing (*n* 311), if dietary information was missing (*n* 171) or implausible (i.e. participants in the highest and lowest 0·5 % of the ratio of energy intake over estimated energy requirement; *n* 392), if there was no informed consent for follow-up (*n* 1605) and if they had a history of CHD or stroke at baseline (self-reported or from linkage; *n* 1114). This leaves 36 418 participants for the present analyses. As these are secondary analysis based on an existing large cohort with long follow-up time, the justification for the sample size is not required.

### Assessment of socio-economic status

The highest obtained educational level was used as a measure of SES. Other SES indicators such as occupation and income were unfortunately not available in this cohort. Educational level was self-reported in the general questionnaire at baseline. The cohort was divided into three groups: low educational level (attended primary school only), medium educational level (attended secondary school or lower and intermediate vocational education) and high educational level (higher vocational education or university).

### Assessment of dietary intake

Dietary intake was assessed at baseline using a self-administered, semi-quantitative FFQ^(^ ^20^ ^)^. This FFQ allows the estimation of the average daily consumption of 178 food items during the preceding year. Energy intake and daily nutrient intakes were estimated by combining the FFQ data with composition data from the Dutch Food Consumption Table^(^ ^21^ ^)^. Relative validity was assessed in a sub-sample of the cohort by comparing the data collected from the questionnaire with data drawn from twelve 24-h recalls. Median 12-month reproducibility of food groups and nutrients ranged from 0·45 to 0·92, whereas median validity was 0·61 for men and 0·53 for women^(^ ^20^ ^,^ ^22^ ^)^. The food items were grouped into thirty-seven food groups as described by Biesbroek *et al.* ^(^ ^23^ ^)^. These food groups were based on twenty-three main standard Dutch food groups, and an additional breakdown of some groups into relevant subgroups. Energy intake per food group divided by total energy intake (energy percentage) was used in the analyses.

### Ascertainment of incident CHD and stroke

Data on morbidity were obtained through linkage with the hospital discharge diagnoses register of the Dutch Hospital Association and Order of Medical Specialists by a validated probabilistic method^(^ ^19^ ^)^. Information on vital status was obtained through linkage with the municipal population registries. Causes of death were obtained through linkage with the Cause of Death Registry from Statistics Netherlands. Participants were followed up for the first occurrence of CHD (International Classification of Diseases (ICD)–9: 410–414, 427·5, 798·1, 798·2 and 798·9; ICD-10: I20–I25, I46 and R96) or stroke (ICD-9: 430–438; ICD-10: I60–I67, I69, G45), either fatal or non-fatal. Follow-up ended on the day of diagnosis, on the day of death, at the end of the study or when a participant was lost to follow-up, whichever came first. The censor date was 31 December 2010.

### Lifestyle and anthropometric variables

The general questionnaire included information on physical activity, smoking habits and disease history. At baseline, a physical examination including measurement of body weight, height and blood pressure was performed and non-fasting blood samples were drawn^(^ ^19^ ^)^. BMI was calculated as weight (kg)/height (cm)^2^. Physical activity was assessed using a short questionnaire and classified according to the validated Cambridge Physical Activity Index^(^ ^24^ ^,^ ^25^ ^)^. Missing values for physical activity (14·2 % of the population) were imputed in the EPIC-NL cohort by multiple imputation using five imputed data sets^(^ ^26^ ^)^. PROC MI with the Fully Conditional Specification method to impute missing data on categorical variables with an arbitrary missing data pattern was used^(^ ^27^ ^)^. Smoking was categorised as current, never or former smoker. Baseline status of hypertension, hyperlipidaemia and diabetes was based on self-report.

### Statistical analyses

Baseline characteristics were tabulated by educational level. First, to obtain the population-based dietary patterns, the thirty-seven food groups were entered into a principal component analysis (PCA) with varimax rotation. The food groups expressed in energy percentage were directly used in the PCA. The number of factors to retain was determined by use of the scree plot, Eigenvalues and the interpretability of the factors. Component loadings of the food groups higher than 0·20 or lower than −0·20 were considered relevant for a factor. Using Generalised Linear Models, mean factor scores by educational level were estimated after adjustment for age, sex and cohort. Second, PCA was done in a similar way as described above for each of the three educational groups. Similarity of the obtained dietary patterns was evaluated by comparing the factor loadings of the food groups that contributed to the obtained patterns between the three groups.

For each derived dietary pattern in the whole population, the corresponding factor scores were divided into quartiles. Participants in the highest quartile (Q4) were considered to be high adherents to that pattern. For every dietary pattern, the mean food group and nutrient intakes of the high adherents were compared over the educational groups using Generalised Linear Models to adjust for age, sex and cohort to account for differences in the distribution of these variables.

Hazard ratios (HR) for the association between the dietary patterns derived in the whole population and incident CHD and stroke were obtained from Cox proportional hazard models. The results of these analysis based on the patterns derived in the whole population were presented for both the whole population and stratified by educational level. HR were shown continuously per standard deviation increase in the factor score. To evaluate whether the association of dietary patterns with CHD and stroke differed significantly according to strata of educational level, interaction terms between the dietary pattern scores and education groups were included in the Cox proportional hazard models. All Cox proportional hazard models included the sub-cohorts as strata to allow for different baseline hazard functions in EPIC-Prospect and EPIC-MORGEN. The first model was adjusted for sex and age, whereas the second model additionally included smoking status, physical activity and energy intake as potential confounders in the diet–disease association. These were chosen based on their known association with both CVD and SES^(^ ^28^ ^–^ ^30^ ^)^. To account for the multiple imputation of missing data on the confounders in the second model, PROC MYANALYZE was used to combine the different HR from the imputed data sets into one overall estimate. All statistical analyses were performed using SAS software (version 9.4; SAS Institute Inc.). A two-sided *P* value of <0·05 was considered statistically significant.

The EPIC-Prospect cohort exists of older women only, who more often have a lower educational level (46 %) compared with women of the EPIC-MORGEN cohort (36 %). Therefore, sensitivity analyses were performed to derive the educational level-specific dietary patterns by cohort. The derived dietary patterns were reasonably comparable with those of the whole EPIC-NL cohort (based on factor loadings; results not shown). Therefore, the results are presented for the whole cohort.

## Results

The low-educated group consisted of 14 331 participants (39·4 %); 14 632 participants (40·2 %) had a medium education; and 7455 participants (20·5 %) had a high educational level (Table 1). A higher proportion of the high-educated group was male, and on average they were about 6 years younger and had a two-point-lower BMI than the low-educated group. At baseline, self-reported hypertension, hyperlipidaemia and diabetes were more prevalent in the low-educated group. Food and nutrient intake by educational level is presented in the online Supplementary Table S1. A higher education was associated with lower total energy intake and higher intakes of food items that are considered to be part of a healthy diet, such as fruits, raw vegetables, fish, high-fibre cereals and oils/diet margarines, in combination with lower intakes of meat, French fries, high-fat dairy and sugar-containing soft drinks.

**Table 1:** Baseline characteristics according to educational level in European Prospective Investigation into Cancer and Nutrition-Netherlands (Mean values and standard deviations; percentages and frequencies; mean values with their standard errors)

	Low education (*n* 14 331)		Medium education (*n* 14 632)		High education (*n* 7455)	
Characteristics	Mean or %	sd or frequency	Mean or %	sd or frequency	Mean or %	sd or frequency
Cohort: prospect	50·7	7266	41·1	6015	34·8	2596
Age (years)	52·4	10·5	47·2	12·8	46·5	11·0
Male (sex)	21·0	3002	25·2	3686	33·6	2501
BMI (kg/m^2^)*	26·7	4·2	25·3	3·8	24·4	3·3
Smoking†						
Former	28·5	4079	32·1	4699	34·5	2566
Current	32·7	4684	30·1	4409	26·5	1971
Physical activity						
Moderately inactive	24·3	3475	25·2	3686	24·5	1829
Moderately active	23·4	3353	26·7	3912	29·8	2224
Active	43·4	6216	41·5	6065	40·2	2997
Hypertension	24·1	3449	20·2	2953	17·8	1327
Hyperlipidaemia	9·2	1318	7·2	1052	6·63	494
Diabetes	2·6	377	1·5	212	0·8	59
Dietary pattern factor scores‡						
‘Western pattern’						
Mean	0·01		0·03		−0·09	
se	0·007		0·006		0·009	
‘Prudent pattern’						
Mean	−0·18		0·06		0·24	
se	0·008		0·007		0·011	
‘Traditional pattern’						
Mean	0·34		−0·08		−0·49	
se	0·008		0·008		0·011	

* *n* 8, *n* 8 and *n* 3 missing for low, medium and high education, respectively.

† *n* 8, *n* 7 and *n* 8 missing for low, medium and high education, respectively.

‡ Adjusted for age, sex and cohort.

### Whole-cohort-based dietary patterns

**Fig. 1:**
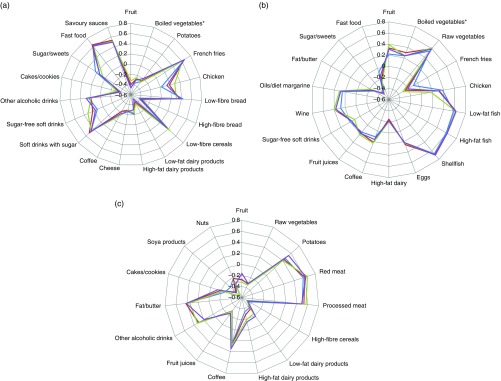
Radar charts of the component loadings of the ‘Western’ (a), ‘prudent’ (b) and ‘traditional’ (c) dietary pattern derived through principal component analysis per educational group and in the total population. Only food groups with a factor loading >0·20 or <−0·20 in at least one of the educational groups are presented. * Includes legumes. 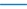, Low education; 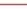, medium education; 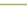, high education; 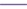, total population.

The whole cohort PCA derived three patterns that had Eigenvalues above 2, whereas for the subsequent patterns these were between 0·4 and 1·6 (online Supplementary Fig. S1(A)). The derived patterns could be labelled as ‘Western’, ‘prudent’ and ‘traditional’. The factor loadings of the food groups significantly contributing to the dietary patterns, that is factor loadings higher than 0·20 or lower than −0·20, are presented in Fig. 1. The ‘Western’ pattern was characterised by a high contribution of French fries, savoury snacks, savoury sauces, sugar-containing soft drinks, low-fibre cereals, other alcoholic drinks and processed meat and a low consumption of fruit, dairy, high-fibre bread and vegetables (online Supplementary Table S2). The ‘prudent’ pattern was characterised by a high consumption of (shell-) fish, vegetables, wine, fruit, oils/diet margarines and eggs and a low consumption of sugar/sweets, French fries, fat/butter and high-fat dairy products (online Supplementary Table S3). The ‘traditional’ pattern was characterised by a high consumption of red meat, processed meat, potatoes, fat/butter, coffee/tea, boiled vegetables and eggs and a low consumption of soya products, high-fibre cereals, fruit juice, raw vegetables and nuts (online Supplementary Table S4).

A higher factor score indicates better adherence to the dietary pattern. The mean ‘Western’ pattern score was highest in the medium-educated group (0·03) and lowest in the high-educated group (−0·09) (Table 1). With higher educational levels, the mean factor score for the ‘prudent’ pattern increased, whereas it decreased for the ‘traditional’ pattern after adjustment for sex, age and cohort. The most distinctive difference in mean factor score was observed between the groups with a high (−0·49) and a low (0·34) education for the ‘traditional pattern’. These findings illustrate that there is a difference in the adherence to these patterns between groups with a different educational level.

### Education-specific dietary patterns

To investigate whether there are education-specific dietary patterns, a PCA was performed for each educational group separately. In all educational groups, the first three PCA patterns had Eigenvalues above 2, whereas the Eigenvalues for the subsequent patterns varied between 0·5 and 1·5 (online Supplementary Fig. S1(B)–(D)). The factor loadings for all food groups are presented in the online Supplementary Tables S2–S4. The overlapping lines in Fig. 1 show that for most relevant food groups the factor loadings were similar in magnitude across educational levels and similar to the factor loadings of the dietary patterns derived in the whole population. Therefore, also in each educational group the three patterns were labelled ‘Western’, ‘prudent’ and ‘traditional’. Because the dietary patterns across the educational groups were essentially the same as those derived in the whole population, we continued our analysis with the patterns derived in the whole population.

### Food intake of adherents (Q4) to whole-cohort-derived dietary patterns according to educational level

In Q4 of the ‘Western’ pattern, low-educated participants had a higher consumption of French fries, low-fibre bread, sugar-containing soft drinks and processed meat compared with high-educated participants (online Supplementary Table S5). Consumption of food items with negative factor loadings on the ‘Western’ pattern, for example high-fibre bread, low-fat dairy products and cheese, was lower for participants in Q4 with a low education.

With increasing educational level, the consumption of shellfish, oils/diet margarines, wine, raw vegetables and fruit increased significantly in high adherents to the ‘prudent’ pattern (Q4, online Supplementary Table S6). Low-educated adherents to this pattern consumed more of other food groups that scored positive on this pattern, such as boiled vegetables, chicken and eggs. The intake of most food groups that scored negative on the ‘prudent’ pattern – that is French fries, high-fat dairy and fat/butter – was lower in adherents with a high education than in adherents with a low education.

High adherents to the ‘traditional’ pattern with a low education consumed more potatoes and fat/butter than those with a high education (online Supplementary Table S7). Except for low-fibre cereals, fruit juices and savoury snacks, mean intake of all food groups inversely associated with the ‘traditional’ pattern was lower for adherents with a low education. For all dietary patterns, these small differences in food intake resulted in different nutrient intake as well (online Supplementary Tables S5–S7).

### Dietary pattern–disease associations

The observed educational differences in the factor scores may possibly affect dietary pattern–disease associations. Neither in the educational groups nor in the total study sample associations were observed between adherence to the ‘Western’ dietary pattern and the incidence of CHD or stroke after adjustment for possible confounders. Higher adherence to the ‘prudent’ dietary pattern was significantly associated with incident fatal and non-fatal CHD in participants with a medium education only (HR per sd: 1·10; 95 % CI 1·02, 1·18; Table 2). In none of the educational groups associations between the ‘prudent’ dietary pattern and the incidence of stroke were observed. Higher adherence to the ‘traditional’ pattern was associated with an increased incidence of CHD and stroke after adjustment for age and sex in all three educational groups. Further adjustment attenuated the associations and only in the low-educated group the association remained statistically significant. The point estimates were in the same direction for the medium- and high-educated groups. Despite these differences, none of the interaction terms investigating whether the associations between dietary patterns and disease risk differed by educational level were statistically significant; *P* values varied between 0·21 and 0·98.

**Table 2:** Association between principal component analysis (PCA)-derived dietary patterns and incident CHD and stroke* (Adjusted hazard ratios (HR) and 95 % confidence intervals)

			Stratified by educational level (continuous (per sd))						
	Continuous (per sd)		Low education		Medium education		High education		*P*_interaction_
	HR	95 % CI	HR	95 % CI	HR	95 % CI	HR	95 % CI	(SES×dietary pattern score)
‘Western’									
CHD									
Model 1	1·06	1·00, 1·12	1·02	0·95, 1·10	1·14	1·04, 1·25	1·05	0·91, 1·22	0·41
Model 2	1·00	0·95, 1·05	0·96	0·89, 1·03	1·09	0·99, 1·19	1·00	0·86, 1·16	0·21
Stroke									
Model 1	1·09	1·01, 1·18	1·06	0·95, 1·18	1·19	1·05, 1·35	0·95	0·77, 1·18	0·36
Model 2	1·03	0·95, 1·12	1·00	0·90, 1·12	1·12	0·98, 1·27	0·91	0·73, 1·12	0·43
‘Prudent’									
CHD									
Model 1	1·05	1·01, 1·09	1·03	0·97, 1·09	1·10	1·03, 1·18	1·01	0·91, 1·13	0·98
Model 2	1·02	0·98, 1·07	0·99	0·93, 1·06	1·10	1·02, 1·18	1·01	0·89, 1·13	0·74
Stroke									
Model 1	0·99	0·94, 1·05	1·03	0·95, 1·12	0·95	0·86, 1·04	0·97	0·94, 1·14	0·61
Model 2	0·96	0·90, 1·02	1·01	0·93, 1·11	0·91	0·82, 1·01	0·90	0·77, 1·07	0·44
‘Traditional’									
CHD									
Model 1	1·15	1·10, 1·20	1·18	1·11, 1·25	1·09	1·01, 1·18	1·12	1·00, 1·27	0·33
Model 2	1·09	1·04, 1·14	1·11	1·04, 1·18	1·05	0·97, 1·14	1·10	0·97, 1·25	0·48
Stroke									
Model 1	1·15	1·08, 1·22	1·18	1·08, 1·29	1·12	1·01, 1·24	1·13	0·96, 1·33	0·40
Model 2	1·09	1·02, 1·16	1·12	1·02, 1·22	1·05	0·95, 1·18	1·09	0·92, 1·28	0·47

* Model 1: adjusted for age and sex (and educational level in the whole population). Model 2: model 1 with additional adjustment for smoking status, physical activity and kJ (kcal).

## Discussion

Our study found that dietary patterns, derived with PCA when stratified by educational level, were similar to those derived in the whole population. On the basis of the scree plots, Eigenvalues and factor loadings of the food groups, three dietary patterns were observed that could be labelled as ‘Western’, ‘prudent’ and ‘traditional’. For the whole-population-based patterns, we observed that the mean factor score for the ‘Western pattern’ was similar for the low- and middle-educated groups but lower for the high-educated group. With higher education, the mean score on the ‘prudent pattern’ increased, whereas it decreased for the ‘traditional pattern’. The observed differences in distribution of the dietary factor scores emphasise the existing association between education and dietary intake and translate into differences in food and nutrient intake among high adherents to the patterns (those in Q4 of the factor score). In general, illustrated by the higher factor loadings with higher education, high adherents to the ‘prudent’ pattern consumed more of the foods that are positively associated with this pattern and less of some foods that are negatively associated. The consumption of some of the food groups positively associated with the ‘Western’ and the ‘traditional’ pattern was higher in participants of Q4 with a low and middle education compared with those with a high education. Differences in nutrient intake resulting from different food consumption within the same highest quartile of the dietary patterns were often statistically significant and often in favour of those with a higher education. After adjustments for several baseline characteristics, no obvious differences in associations between dietary patterns and the incidence of fatal and non-fatal CHD or stroke were observed between the different educational groups.

In many studies it is noted that there is a clear link between SES and diet quality. This is summarised in a review article by Darnon and Drewnowski that stated that the consumption of whole grains, lean meats, fish, low-fat dairy products and fresh vegetables and fruit is consistently associated with higher SES, whereas the consumption of fatty meats, refined grains and added fats is associated with lower SES^(^ ^31^ ^)^. In our study, we observed that although the dietary patterns itself were quite similar (importance of the food groups) the underlying association between education and dietary patterns scores resulted in lower factor scores for the ‘Western’ and ‘traditional’ patterns and higher scores for the ‘prudent’ pattern with higher education. Therefore, in high adherents to the pattern (Q4 of the factor score) with higher education, we observed higher consumption of foods that are considered to be part of a healthy diet, regardless of the dietary pattern under study. A possible explanation might be that the lower-educated individuals encounter material hardship (more financial constraints and limited access to /availability of healthy foods) than those with a high education, and therefore consume less of the healthy foods^(^ ^32^ ^)^. This is also supported by the above-mentioned review article that showed that the lowest-cost diets were also the least healthy, and that low-cost foods had higher energy density and had less nutritional content^(^ ^31^ ^)^.

Even though the dietary patterns itself are very similar, it is important to keep in mind that SES is still a clear and important determinant of lifestyle and diet. A ‘prudent’ dietary pattern is more often found in high-educated individuals, whereas a ‘Western’ pattern is more likely to occur at a low educational level^(^ ^9^ ^,^ ^11^ ^,^ ^16^ ^)^. Therefore, policymakers should focus on developing interventions that reduce inequalities, but based on our results there is no need to focus such interventions on dietary patterns that are observed specifically for groups with a certain educational level.

In a previous paper with data from the EPIC-NL cohort^(^ ^23^ ^)^, we already published that adherence to the *a posteriori*-derived ‘Western’ or ‘prudent’ dietary patterns was not associated with CHD risk in the whole population, whereas adherence to the ‘traditional’ pattern was significantly associated with an increased risk of CHD. The current study adds to these findings that there is no clear indication for interaction by educational level in the dietary pattern–disease association. After stratification by educational group, only the ‘traditional’ pattern in the low-educated group remains statistically significant, whereas in the higher-educated groups the association is positive but not statistically significant. This might be because of a lack of power, as the number of participants differs largely between the educational groups (*n* 5438, 2870 and 795 for, respectively, low, medium and high education).

A potential limitation of the study is that only self-reported educational level is available to define SES. A composite measure of financial income and education might better represent SES^(^ ^33^ ^)^. However, education itself is a reasonable proxy of SES and is associated with (risk factors of) CVD^(^ ^33^ ^,^ ^34^ ^)^. In addition, we only had baseline data on dietary intake. In a small sample of EPIC-MORGEN, 40 % of participants were consistently assigned to the same dietary pattern for three surveys with a 5-year interval each^(^ ^35^ ^)^. This may have affected the dietary pattern–disease associations if participants during follow-up changed from an unhealthy pattern to a healthier one or vice versa. The strength of our study is its size, which made it possible to split the cohort into three educational groups and still have an adequate number of participants in each group. We used PCA to distinguish *a posteriori* food patterns. Central in this method is the choosing of how many factors are meaningful to retain. This is somewhat arbitrary and thus can lead to different final patterns^(^ ^36^ ^)^. This partly explains why some studies report only two patterns^(^ ^37^ ^)^, whereas others report five to six dietary patterns^(^ ^38^ ^,^ ^39^ ^)^.

To conclude, this study found that dietary patterns are similar over different educational groups and do not differ from patterns observed in the total population. In both the total population and in the low-, medium- and high-educated participants, a ‘Western’, ‘prudent’ and ‘traditional’ dietary pattern was observed. As a result of the association between educational level and dietary pattern score, within Q4 of the whole-population-derived patterns (the participants that are considered to adhere to a pattern), small differences in the consumption of food groups were observed and it seemed more favourable in those with a higher education. No statistically significant differences in associations between dietary patterns and the incidence of CHD or stroke between the educational groups were observed after adjusting for baseline risk factors.

## Supplementary material

For supplementary material accompanying this paper visit http://dx.doi.org/10.1017/S0007114518000569. click here to view supplementary material
