# Supplementary material for: Dietary patterns within educational groups and their association with CHD and stroke in the European Prospective Investigation into Cancer and Nutrition-Netherlands cohort
Source: Br J Nutr. 2018 Apr 28;119(8):949–56. doi: 10.1017/S0007114518000569 (PMC6088537; doi:10.1017/S0007114518000569)
Supplement: Supplementary file 1 [file S0007114518000569sup.zip › S0007114518000569sup003.docx]

**ONLINE SUPPORTING MATERIAL**

**Dietary patterns within educational groups and their association with coronary heart disease and stroke in the EPIC-NL cohort**

**Sander Biesbroek^1^, Mirjam C Kneepkens^1^, Saskia W. van den Berg^1^, Heidi P. Fransen^2^, Joline W. Beulens^2,3^, Petra H.M. Peeters^2,4^, and Jolanda M.A. Boer^1^.**

1. National Institute for Public Health and the Environment, Bilthoven, the Netherlands
2. Julius Center for Health Sciences and Primary Care, University Medical Center Utrecht, Utrecht, the Netherlands.
3. Department of Epidemiology & Biostatistics, EMGO+ Institute for Health and Care Research, VU University Medical Center, Amsterdam, The Netherlands
4. School of Public Health, Imperial College London, London, United Kingdom

**Correspondence:**

Sander Biesbroek, Centre for Nutrition, Prevention and Health Services, National Institute for Public Health and the Environment (RIVM), PO Box 1, 3720BA Bilthoven, The Netherlands, E-mail: sander.biesbroek@rivm.nl.

**Supplemental table 2.** Component loadings of the ‘Western’ pattern derived with Principal Component Analyses per educational group and in the whole cohort.*

| **Food group factor loadings** | **Low education**  **(*n*=14331)** | **Medium education**  **(*n*=14632)** | **High education**  **(*n*=7455)** | **EPIC-NL** |
| --- | --- | --- | --- | --- |
| French fries | 0.66 | 0.65 | 0.61 | 0.65 |
| Potatoes | -0.27 | -0.26 | -0.29 | -0.20 |
| Coffee/tea | -0.23 | -0.23 | -0.12 | -0.17 |
| Sugar-free soft drinks | 0.12 | 0.22 | 0.32 | 0.16 |
| Sugar containing soft drinks | 0.47 | 0.51 | 0.46 | 0.46 |
| Fruit juices | -0.08 | 0.02 | 0.09 | -0.07 |
| Wine | -0.01 | -0.02 | 0.02 | 0.04 |
| Other alcoholic drinks | 0.23 | 0.18 | 0.18 | 0.28 |
| Low-fiber bread | 0.43 | 0.36 | 0.30 | 0.38 |
| High-fiber bread | -0.43 | -0.39 | -0.35 | -0.37 |
| Egg | 0.01 | 0.03 | 0.05 | 0.04 |
| Fruit | -0.45 | -0.40 | -0.33 | -0.47 |
| Cakes/cookies | -0.33 | -0.37 | -0.31 | -0.39 |
| Low-fiber cereals | 0.36 | 0.42 | 0.44 | 0.37 |
| High-fiber cereals | -0.04 | -0.05 | -0.04 | -0.12 |
| Boiled vegetables/legumes | -0.36 | -0.28 | -0.24 | -0.30 |
| Raw vegetables | -0.09 | -0.08 | -0.04 | -0.13 |
| Cheese | -0.26 | -0.29 | -0.28 | -0.26 |
| Low-fat dairy products | -0.44 | -0.44 | -0.40 | -0.51 |
| High-fat dairy products | -0.21 | -0.23 | -0.24 | -0.30 |
| Soy products | 0.06 | -0.01 | -0.04 | -0.06 |
| Nuts | 0.17 | 0.12 | 0.09 | 0.10 |
| Savoury snacks | 0.62 | 0.64 | 0.60 | 0.61 |
| Soups | -0.02 | -0.04 | -0.04 | -0.03 |
| Sugar/sweets | 0.23 | 0.13 | 0.06 | 0.13 |
| Oils/diet margarine | -0.09 | -0.07 | -0.03 | -0.08 |
| Fat/butter | 0.03 | -0.06 | -0.13 | 0.04 |
| Savoury sauces | 0.51 | 0.53 | 0.51 | 0.49 |
| Low-fat fish | -0.13 | -0.08 | -0.05 | -0.12 |
| High-fat fish | 0.02 | 0.06 | 0.13 | 0.05 |
| Shellfish | 0.09 | 0.09 | 0.14 | 0.09 |
| Red meat | -0.04 | 0.08 | 0.14 | 0.14 |
| Processed meat | 0.15 | 0.11 | 0.12 | 0.22 |
| Chicken | 0.02 | 0.18 | 0.29 | 0.13 |

*: in green are food groups positively associated with the factor >0.20; in red are food groups inversely associated with the factor <-0.20.
