# Supplementary material for: Dietary patterns within educational groups and their association with CHD and stroke in the European Prospective Investigation into Cancer and Nutrition-Netherlands cohort
Source: Br J Nutr. 2018 Apr 28;119(8):949–56. doi: 10.1017/S0007114518000569 (PMC6088537; doi:10.1017/S0007114518000569)
Supplement: Supplementary file 1 [file S0007114518000569sup.zip › S0007114518000569sup006.docx]

**ONLINE SUPPORTING MATERIAL**

**Dietary patterns within educational groups and their association with coronary heart disease and stroke in the EPIC-NL cohort**

**Sander Biesbroek^1^, Mirjam C Kneepkens^1^, Saskia W. van den Berg^1^, Heidi P. Fransen^2^, Joline W. Beulens^2,3^, Petra H.M. Peeters^2,4^, and Jolanda M.A. Boer^1^.**

1. National Institute for Public Health and the Environment, Bilthoven, the Netherlands
2. Julius Center for Health Sciences and Primary Care, University Medical Center Utrecht, Utrecht, the Netherlands.
3. Department of Epidemiology & Biostatistics, EMGO+ Institute for Health and Care Research, VU University Medical Center, Amsterdam, The Netherlands
4. School of Public Health, Imperial College London, London, United Kingdom

**Correspondence:**

Sander Biesbroek, Centre for Nutrition, Prevention and Health Services, National Institute for Public Health and the Environment (RIVM), PO Box 1, 3720BA Bilthoven, The Netherlands, E-mail: sander.biesbroek@rivm.nl.

**Supplemental Table 6.** Mean dietary intake in Q4 of the ‘western’ dietary pattern stratified by educational level.

|  | **‘Western’ pattern adherents with a:** | | |  |
| --- | --- | --- | --- | --- |
|  | **Low education**  **N=3043** | **Medium education**  **N=4173** | **High education**  **N=1889** | **P-value**  **(High vs Low education)** |
| **Food groups (en%)** |  |  |  |  |
| *Positive factor loadings* |  |  |  |  |
| French fries | 5.9 (0.07) | 4.9 (0.06) | 4.2 (0.09) | <0.0001 |
| Sugar containing soft drinks | 2.7 (0.04) | 2.4 (0.04) | 1.7 (0.06) | <0.0001 |
| Other alcoholic drinks | 4.5 (0.11) | 4.9 (0.10) | 4.8 (0.14) | 0.3797 |
| Low-fiber bread | 8.5 (0.12) | 7.0 (0.10) | 5.5 (0.15) | <0.0001 |
| Low-fiber cereals | 3.0 (0.07) | 3.8 (0.06) | 4.2 (0.09) | <0.0001 |
| Savoury snacks | 4.8 (0.06) | 5.0 (0.05) | 5.7 (0.07) | <0.0001 |
| Savoury sauces | 3.0 (0.04) | 3.1 (0.03) | 3.3 (0.05) | 0.0001 |
| Processed meat | 4.8 (0.06) | 4.1 (0.05) | 3.5 (0.08) | <0.0001 |
|  |  |  |  |  |
| *Negative factor loadings* |  |  |  |  |
| Potatoes | 3.6 (0.04) | 2.8 (0.03) | 2.2 (0.04) | <0.0001 |
| High-fiber bread | 7.7 (0.11) | 8.9 (0.10) | 10.1 (0.14) | <0.0001 |
| Cakes/cookies | 3.1 (0.05) | 3.4 (0.04) | 3.6 (0.07) | 0.0039 |
| Boiled vegetables/legumes | 1.3 (0.01) | 1.2 (0.01) | 1.1 (0.02) | <0.0001 |
| Cheese | 4.0 (0.07) | 4.4 (0.06) | 5.1 (0.08) | <0.0001 |
| Low-fat dairy products | 3.1 (0.05) | 3.3 (0.04) | 3.4 (0.06) | 0.0004 |
| High-fat dairy products | 4.6 (0.06) | 4.4 (0.05) | 4.1 (0.08) | <0.0001 |
|  |  |  |  |  |
| **Macronutrient intake** |  |  |  |  |
| Energy (kJ) | 9975 (45) | 9605 (39) | 9396 (57) | <0.0001 |
| Energy density (kJ/gram) | 3.40 (0.01) | 3.29 (0.01) | 3.23 (0.02) | <0.0001 |
| Protein (en%) |  |  |  |  |
| Vegetable | 5.3 (0.02) | 5.4 (0.02) | 5.6 (0.02) | <0.0001 |
| Animal | 8.8 (0.04) | 8.8 (0.03) | 8.7 (0.05) | 0.1360 |
| Fat (en%) |  |  |  |  |
| Saturated | 14.9 (0.05) | 14.3 (0.04) | 14.1 (0.06) | <0.0001 |
| Monounsaturated | 15.0 (0.04) | 14.5 (0.04) | 14.5 (0.06) | <0.0001 |
| Polyunsaturated | 7.1 (0.03) | 7.1 (0.03) | 7.2 (0.04) | 0.3531 |
| Carbohydrates (en%) |  |  |  |  |
| Mono/disaccharides | 20.7 (0.11) | 20.6 (0.09) | 20.0 (0.14) | 0.0002 |
| Polysaccharides | 24.1 (0.09) | 23.9 (0.08) | 23.6 (0.11) | 0.0001 |
| Fiber (gr/day) | 22.7 (0.12) | 22.3 (0.10) | 22.5 (0.15) | 0.3925 |
| Alcohol (gr/day) | 13.5 (0.35) | 16.6 (0.30) | 19.6 (0.30) | <0.0001 |

Adjusted for age, sex and cohort. Only food groups with factor loadings >0.20 or <-0.20 are presented. Food groups, mean (SE), are expressed as percentage contribution to total daily energy intake.
